# Expression of oestrogen and progesterone receptors in low-grade endometrial stromal sarcomas

**DOI:** 10.1054/bjoc.1999.1038

**Published:** 2000-02-01

**Authors:** O Reich, S Regauer, W Urdl, M Lahousen, R Winter

**Affiliations:** Departments of Obstetrics & Gynecology and Pathology, University of Graz, Auenbruggerplatz, Graz, A-8036, Austria

**Keywords:** uterus, cancer, endometrium, stromal sarcoma, oestrogen receptor, progesterone receptor, immunohistochemistry, treatment

## Abstract

We analysed oestrogen receptor (ER) and progesterone receptor (PR) expression in a retrospective series of 21 low-grade endometrial stromal sarcomas (LGSSs). Archival formalin-fixed and paraffin-embedded material was analysed by immunohistochemistry. ER and PR were measured with monoclonal antibodies and the peroxidase-antiperoxidase method and a score was calculated as for breast carcinoma based on both the percentage of positive tumour cell nuclei and the staining intensity. ER were seen in 15 (71%) and PR in 20 (95%) of tumours respectively. ER expression was scored as high in three (14%), moderate in four (19%), and low in eight (38%) tumours. Six (29%) tumours did not stain for ER and all of these were positive for PR. PR expression was scored as high in eight (38%), moderate in ten (47%) and weak in two (10%) LGSSs. Only one (5%) LGSS did not stain for PR (this tumour was positive for ER). ER and PR expression in LGSS is heterogeneous. This may have implications for hormone therapy in the management of these tumours. These results suggest that ER and PR should be routinely quantified in LGSSs by immunohistochemical methods. © 2000 Cancer Research Campaign


British Journal of Cancer (2000) **82**(5), 1030–1034
© 2000 Cancer Research Campaign
DOI: 10.1054/ bjoc.1999.1038, available online at http://www.idealibrary.com on IDEAL®

### Expression of oestrogen and progesterone receptors in low-grade endometrial stromal sarcomas

**O Reich[1], S Regauer[2], W Urdl[1], M Lahousen[1] and R Winter[1]**

Departments of [1]Obstetrics & Gynecology and [2]Pathology, University of Graz, Auenbruggerplatz, A-8036 Graz, Austria

**Summary** We analysed oestrogen receptor (ER) and progesterone receptor (PR) expression in a retrospective series of 21 low-grade endometrial stromal sarcomas (LGSSs). Archival formalin-fixed and paraffin-embedded material was analysed by immunohistochemistry. ER and PR were measured with monoclonal antibodies and the peroxidase-antiperoxidase method and a score was calculated as for breast carcinoma based on both the percentage of positive tumour cell nuclei and the staining intensity. ER were seen in 15 (71%) and PR in 20 (95%) of tumours respectively. ER expression was scored as high in three (14%), moderate in four (19%), and low in eight (38%) tumours. Six (29%) tumours did not stain for ER and all of these were positive for PR. PR expression was scored as high in eight (38%), moderate in ten (47%) and weak in two (10%) LGSSs. Only one (5%) LGSS did not stain for PR (this tumour was positive for ER). ER and PR expression in LGSS is heterogeneous. This may have implications for hormone therapy in the management of these tumours. These results suggest that ER and PR should be routinely quantified in LGSSs by immunohistochemical methods. © 2000 Cancer Research Campaign

**Keywords:** uterus; cancer; endometrium; stromal sarcoma; oestrogen receptor; progesterone receptor; immunohistochemistry; treatment

Low-grade endometrial stromal sarcomas (LGSSs) are rare, accounting for about 0.2% of all genital tract malignancies (Koss et al, 1965), and occur predominantly in premenopausal women (Evans, 1982). Treatment of LGSSs is primarily surgical, and a positive correlation between extent of surgery and recurrence or metastasis has been reported (Krieger and Gusberg, 1973; Gadducci et al, 1996). Recurrences develop in one-third to one-half of patients (Chang et al, 1990; Clement, 1993) and can appear as long as 30 years after initial therapy (Doran, 1908; Gloor, 1982; Styron, 1989). The role of adjuvant radiation and chemotherapy is poorly defined (Gadducci et al, 1996).

Case reports and several small series have shown that uterine and extrauterine LGSSs can express oestrogen receptors (ER) and progesterone receptors (PR) (Gloor et al, 1982; Baker et al, 1984; Piver et al, 1984; Lantta et al, 1984; Sutton et al, 1986; Katz et al, 1987; Keen and Philip, 1989; Tosi et al, 1989; Dunton et al, 1990; Navarro, 1992; Sabini et al, 1992; Fukunaga et al, 1997, 1998) and are sensitive to sex steroid hormones (Pellillo, 1968; Baggish and Woodruff, 1972; Krumholz et al, 1973; Gloor et al, 1982; Baker et al, 1984; Lantta et al, 1984; Piver et al, 1984; Tsukamoto et al, 1985; Katz et al, 1987, Keen and Philip, 1989; Montag and Manert, 1989; Rand and Lowe, 1990; Navarro et al, 1992; Scribiner and Walker, 1998). These observations have been based on biochemical examinations and have not been confirmed in a larger patient cohort. Immunohistochemical quantification of ER and PR, as performed routinely in breast carcinoma, has not been reported for LGSSs.

In the present study we analysed ER and PR expression in a retrospective series of 21 LGSSs. We used the Remmele scoring system (Remmele and Schicketanz, 1993) to quantify ER and PR expression at the cellular level.

### PATIENTS AND METHODS

Twenty-one LGSSs originating between 1983 and 1998 were retrieved from the archives of the Departments of Obstetrics and Gynecology and Pathology of the University of Graz, Austria. Haematoxylin and eosin (H&E)-stained sections were re-evaluated by two pathologists (SR and OR) and staged according to the guidelines of the 1994 WHO classification of tumours of the female genital tract (Scully et al, 1994). All tumours were composed of cells resembling normal endometrial stromal cells and invaded the myometrium and usually its vascular spaces. Mitoses were fewer than 10/10 high-power fields (HPF = objective $\times$ 40) and necrosis was absent. The International Federation of Gynecology and Obstetrics (FIGO) stage for endometrial carcinoma was adapted for LGSS (stage I, tumours limited to the uterus; stage II, involvement of the cervix; stage III, involvement of the pelvis; and stage IV, disease outside the pelvis).

For quantitative immunohistochemical analysis one or two paraffin blocks with representative portions of the tumours were selected and 4-μm thick sections were stained with antibodies to ER (monoclonal, NCL-ER-6F11, Novocastra, UK) and PR (monoclonal, NCL-PGR-1A6, Novocastra, UK). The peroxidase-antiperoxidase method was used to detect the antigens. ER and PR expression was scored by two pathologists (SR and OR) with a scoring system based on both the percentage of positive tumour

*Received 1 March 1999*
*Revised 12 August 1999*
*Accepted 26 August 1999*

*Correspondence to:* O Reich

Part of this work was presented at the XXII International Congress of the International Academy of Pathology, Nice, France, 18–23 October 1998.

cell nuclei and the staining intensity (Remmele and Schicketanz, 1993). The percentage of positive tumour cell nuclei was estimated on a four-tiered scale (< 10% = 1, 11–50% = 2, 51–80% = 3, >81% = 4). A four-tiered scale was also used to score the staining intensity (negative = 0, weak = 1, moderate = 2, strong = 3). The staining intensity was based on the predominant staining intensity in the tumour. The final immunoreactive score was obtained by multiplying the two scores. A final score of 0 was classified as negative, 1–3 as low, 4–6 as moderate and 8–12 as high. Receptor-positive breast carcinoma samples served as positive controls and normal human serum as negative controls.

The mean age of the patients at surgery was 46 years (range 34–79) and only three patients were post-menopausal. Sixteen patients had surgical stage I, four stage III and one stage IV disease. The most common preoperative diagnosis was uterine leiomyomas. Twenty patients underwent total abdominal hysterectomy with bilateral salpingo-oophorectomy. One patient had only exploratory laparotomy. Four patients (nos 2, 9, 14, 16) with stage III disease additionally had an omentectomy and one patient (no. 16) underwent pelvic lymphadenectomy. Four patients (nos 2, 9, 12, 16) received adjuvant chemotherapy, one patient (no. 14) received adjuvant radiation and 16 patients had no further treatment. Four patients were lost to follow-up and the median follow-up of the remaining 17 patients was 6.3 (1–14) years. All patients were followed through the Department of Obstetrics and Gynecology. Three patients (nos 7, 9, 16) developed pelvic recurrences and one patient (no. 10) had distant metastases in the lung and bone. One patient (no. 7) with pelvic recurrences at 2 and 8 years after initial presentation was reoperated and material for ER/PR determination was available. Two patients (nos 10, 14) died of disease and one patient (no. 4) of unrelated causes. None of the patients had hormonally active tumours or conditions such as polycystic ovarian disease or hyperthecosis that might be responsible for prolonged oestrogen stimulation of the endometrium (Press and Scully, 1985).

### RESULTS

Results are summarized in Table 1. ER and PR were strongly expressed in the proliferative phase and early secretory phase of non-tumour endometrial stroma of all premenopausal patients (Figure 1). ER were seen in 15 (71%) and PR in 20 (95%) LGSSs respectively (Figures 2–4). ER expression was scored as high in three (14%), moderate in four (19%), and low in eight (38%) tumours. Six (29%) LGSSs did not stain for ER, all of these tumours were positive for PR. PR expression was scored as high in eight (38%), moderate in ten (47%) and weak in two (10%) tumours. Only one (5%) LGSS did not stain for PR (this tumour was positive for ER).

LGSSs with a high score for ER or PR were uniformly positive throughout the tumour (Figure 2). LGSSs with a moderate score demonstrated variable staining (Figure 3). In LGSSs with a low score the majority of tumour cells were negative. The staining intensity of the positive cells ranged from weakly to strongly positive (Figure 4). The patient (no. 7) with pelvic recurrences 2 and 8 years after initial surgery showed successive loss of ER expression. ER expression was moderate in the primary tumour, low in the first recurrence, and absent in the second. PR expression was high in the primary tumour and both recurrences.

### DISCUSSION

This is the first quantitative immunohistochemical study of ER/PR expression in LGSS. Most tumours expressed ER and PR, but expression of both receptors varied widely ranging from negative to strongly positive.

**Table 1** Expression of ER and PR in 21 patients with LGSS

| Patient No. | Age (at presentation) | Stage (FIGO) | Adjuvant therapy | Clinical follow-up | ER cells | ER SI | ER score | ER Interpretation (overall expression) | PR cells | PR SI | PR score | PR interpretation (overall expression) |
|---|---|---|---|---|---|---|---|---|---|---|---|---|
| 1 | 46 | I | None | NED | 1 | 1 | 1 | Low | 3 | 3 | 9 | High |
| 2 | 50 | III | CT | Lost | 1 | 2 | 2 | Low | 4 | 3 | 12 | High |
| 3 | 74 | I | None | Lost | 0 | 0 | 0 | Negative | 2 | 2 | 4 | Medium |
| 4 | 79 | I | None | DWD | 3 | 3 | 9 | High | 2 | 2 | 4 | Medium |
| 5 | 40 | I | None | NED | 3 | 3 | 9 | High | 2 | 3 | 6 | Medium |
| 6 | 39 | I | None | Lost | 2 | 2 | 4 | Medium | 2 | 2 | 4 | Medium |
| 7 | 44 | I | None | NED | 2 | 2 | 4 | Medium | 4 | 3 | 12 | High |
| 8 | 51 | I | None | NED | 1 | 1 | 1 | Low | 2 | 2 | 4 | Medium |
| 9 | 44 | III | CT | AWD | 4 | 3 | 12 | High | 4 | 3 | 12 | High |
| 10 | 35 | IV | None | DOD | 1 | 2 | 1 | Low | 0 | 0 | 0 | Negative |
| 11 | 38 | I | None | NED | 0 | 0 | 0 | Negative | 1 | 3 | 3 | Low |
| 12 | 35 | I | CT | AWD | 0 | 0 | 0 | Negative | 2 | 3 | 6 | Medium |
| 13 | 50 | I | None | NED | 1 | 1 | 1 | Low | 2 | 3 | 6 | Medium |
| 14 | 65 | III | RT | DOD | 1 | 2 | 2 | Negative | 2 | 2 | 4 | Medium |
| 15 | 45 | I | None | NED | 2 | 2 | 4 | Medium | 3 | 3 | 9 | High |
| 16 | 35 | III | CT | AWD | 1 | 1 | 1 | Low | 4 | 3 | 12 | High |
| 17 | 42 | I | None | NED | 0 | 0 | 0 | Negative | 1 | 2 | 2 | Low |
| 18 | 47 | I | None | NED | 0 | 0 | 0 | Negative | 2 | 3 | 6 | Medium |
| 19 | 34 | I | None | Lost | 1 | 1 | 1 | Low | 4 | 3 | 12 | High |
| 20 | 35 | I | None | NED | 1 | 1 | 1 | Low | 4 | 3 | 12 | High |
| 21 | 35 | I | None | NED | 2 | 2 | 4 | Medium | 2 | 3 | 6 | Medium |

CT: chemotherapy, RT: radiotherapy, NED: no evidence of disease, AWD: alive with recurrent disease, DWD: dead without disease, DOD: dead of disease, Lost: lost to follow-up, Cells: percentage of positive tumour cell nuclei (< 10% = 1; 11–50% = 2; 51–80% = 3; > 81% = 4) SI: staining intensity (negative = 0; weak = 1; moderate = 2; strong = 3), Score: Cells × SI, Interpretation: Score 1–3 as low; 4–6 as medium and 8–12 as high.

*© 2000 Cancer Research Campaign*

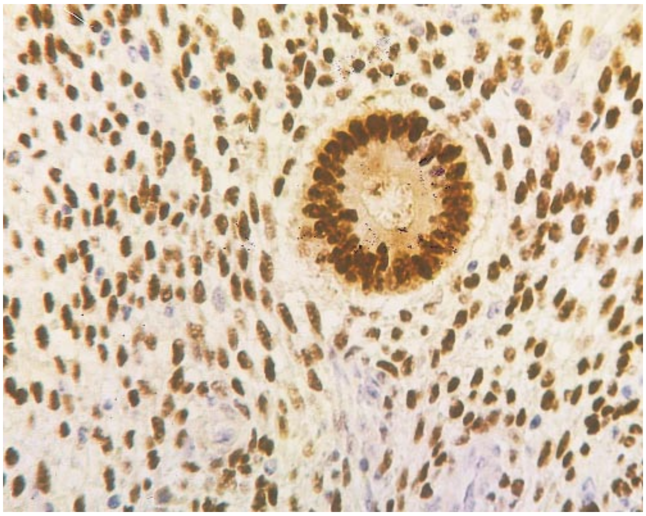

**Figure 1** ER expression in premenopausal nontumour endometrium, proliferative phase. ER are present in the stroma and epithelium of the glands. ER, peroxidase–antiperoxidase, original magnification × 100

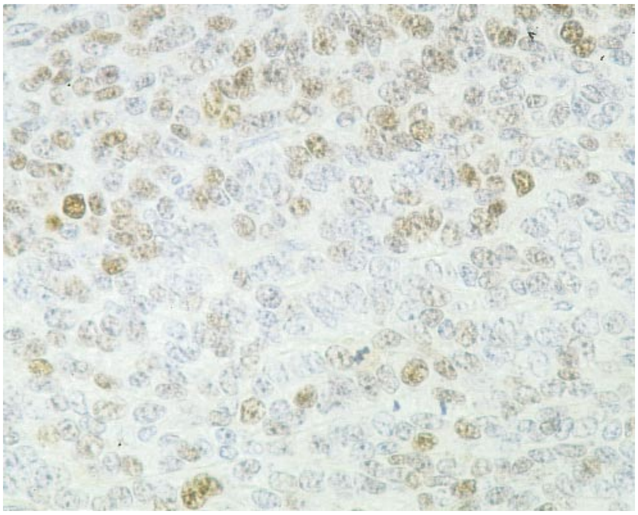

**Figure 3** LGSS with overall medium ER expression. Varying degrees of nuclear staining in the entire specimen indicate heterogeneous receptor expression (patient no. 6, score 4). PR, peroxidase–antiperoxidase, original magnification × 250

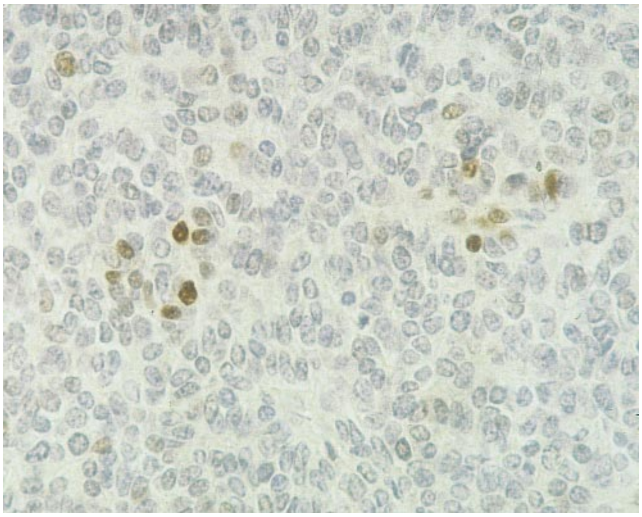

**Figure 2** LGSS with overall low PR expression. Only individual cells or small groups of cells show strong nuclear staining while the majority of cells do not stain (patient no. 17, score 2). PR, peroxidase–antiperoxidase, original magnification × 250

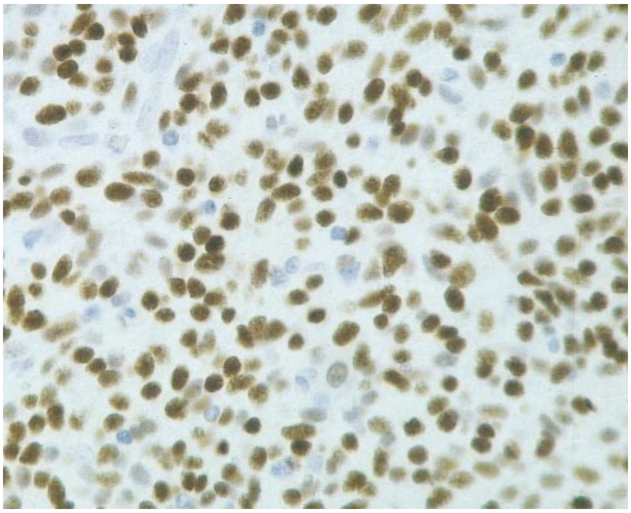

**Figure 4** LGSS with overall high PR expression with homogeneous strong nuclear staining in the absence of cytoplasmatic staining (patient no. 7, score 12). PR, peroxidase–antiperoxidase, original magnification × 200

Oestrogen and progesterone are important regulators of endometrial stromal function and act by binding to their nuclear receptors (Pickartz et al, 1990; Salmi et al, 1998). Most previous studies of sex-steroid receptors in LGSSs were performed biochemically and were based on fewer than five cases of LGSSs (Baker et al, 1984; Lantta et al, 1984; Tsukamoto et al, 1985; Sutton et al, 1986; Katz et al, 1987; Navarro et al, 1992). The reported concentrations of ER in tumour tissue ranged from 0 (Katz et al, 1987) to 1628 (Sutton et al, 1986) and of PR from 0 (Dunton et al, 1990) to 1811 (Dunton et al, 1990) fmol $mg^{-1}$ cytosol protein. However, cytosol-based biochemical methods are not highly specific (Helin et al, 1990). In breast carcinoma, immunohistochemical methods appear more sensitive, as they allow a better prediction of responsivness of the hormone therapy (Robertson et al, 1992). In analogy to breast carcinoma, we were able to demonstrate heterogeneous expression of ER and PR in LGSSs on a cellular level, which suggests different levels of susceptibility to hormonal therapy. Interestingly, in a patient with recurrent disease expression declined successively with each recurrence while PR expression remained high in both recurrences.

Several clinical observations suggest that LGSSs are sensitive to sex-steroid hormones. Katz et al (1987) reported four complete or partial responses with hormonal therapy. All four patients were alive and free of disease or alive with stable disease 2–6 years after initial diagnosis. Regression in size of metastases has been reported after bilateral oophorectomy (Baggish and Woodruff, 1972) and radiation-induced castration (Gloor et al, 1982). A lower incidence of recurrence and longer disease free intervals after bilateral oophorectomy have also been reported (Krieger and Gusberg, 1973). Recurrent disease has been successfully treated with progestins (Pellillo, 1968; Baggish and Woodruff, 1972;

 *© 2000 Cancer Research Campaign*

Krumholz et al, 1973; Gloor et al, 1982; Baker et al, 1984; Tsukamoto et al, 1985; Keen and Philip, 1989; Montag and Manart, 1989; Rad and Lowe, 1990; Horowitz et al, 1996). Scribiner and Walker (1998) reported preoperative treatment of LGSS with progestins. Anecdotal experience by Horowitz et al (1996) suggests that estrogen replacement therapy following a total hysterectomy and bilateral salpingo-oophorectomy in patients with LGSSs may increase the risk for recurrence. While the majority of LGSSs have been shown to contain ER and PR, not all responded to hormonal therapy. Sutton et al (1986) suggested that a critical threshold concentration of sex-steroid receptor is necessary for hormonal manipulation to be successful. The number of patients in our series is too small to analyse for associations between receptor status, hormone treatment and recurrence.

The heterogeneous expression of ER and PR receptors in our study may reflect receptor mutations with antigen loss and heterogeneous tumour populations (Richer et al, 1998). The number of patients in our study is too low to speculate about any association regarding clinical behaviour and receptor status. The three patients with recurrence in our series varied widely in receptor expression. Conceivably, patients with LGSS could benefit from hormonal therapy based on the individual receptor status for primary and recurrent disease. In analogy to breast carcinoma, anti-oestrogens (Luo et al, 1997), selective anti-oestrogen receptor modulators (Cummings et al, 1999), progesterone (Bernoux et al, 1998), anti-progesterone (Eldar-Geva and Healy, 1998), aromatase inhibitors (Miller et al, 1998) and luteinizing hormone-relasing hormone analogues (GNRH-A) (Vignali and Genazzani, 1998) might be useful in this setting. Hormone treatment might benefit patients with advanced disease for preoperative down-staging (Scribiner et al, 1998) or palliation, patients with disease confirmed to the uterus after hysterectomy, or patients with recurrence (Krumholz et al, 1973; Gloor et al, 1982).

In conclusion, we suggest that, as in breast carcinoma, ER and PR should be routinely quantified in LGSSs by immunohistochemical methods.

### ACKNOWLEDGEMENTS

The authors are grateful to Mr Mohamed Al-Effah for exellent technical help and for Dr Karl Tamussino for editing the manuscript.

### REFERENCES

Baggish MS and Woodruff JD (1972) Uterine stromatosis: clinicopathologic features and hormone dependency. *Obstet Gynecol* **40**: 487–498

Baker VV, Walton LA, Fowler WC and Currie JL (1984) Steroid receptors in endolymphatic stromal myosis. *Obstet Gynecol* **63**: 72–74

Bernoux A, De-Cremoux P, Laine-Bidron C, Martin EC, Asselain B and Magdelenat H (1998) Estrogen receptor negative and progesterone receptor positive primary breast cancer: pathological characteristics and clinical outcome. *Breast Canc Res Treat* **49**: 219–225

Chang KL, Crabtree GS, Lim-Tan SK, Kempson RL and Hendrickson MR (1990) Primary uterine endometrial stromal neoplasms. A clinicopathologic study of 117 cases. *Am J Surg Pathol* **14**: 415–438

Clement BC and Young RH (1993) *Tumors and Tumor-like Lesions of the Uterine Corpus and Cervix*. Churchill Livingstone: New York

Cummings SR, Eckert S, Krueger KA, Grady D, Powels TJ, Cauley JA, Norton L, Nickelsen T, Bjarnason NH, Morrow M, Lippmann ME, Black D, Glusman JE, Costa A and Jordan VC (1999) The effect of raloxifen on risk of breast cancer in postmenopausal women. Results from the MORE randomized trial. *JAMA* **281**: 2189–2197

Dunton CJ, Kelsten ML, Brooks SE, Viglione MJ, Carlson JA and Mikuta J (1990) DNA flow cytometric analysis and progesterone receptor data. *Gynecol Oncol* **37**: 268–275

Eldar-Geva T and Healy DL (1998) Other medical management of uterine fibroids. *Baillières Clin Obstet Gynaecol* **12**: 269–288

Evans HL (1982) Endometrial stromal sarcoma and poorly differentiated endometrial sarcoma. *Cancer* **50**: 2170–2182

Fukunaga M, Miyazawa Y and Ushigoma S (1997) Endometrial stromal sarcoma with ovarian sex cord-like differentiation. *Pathol Int* **47**: 412–415

Fukunaga M, Ishihara A and Ushigome S (1998) Extrauterine low-grade endometrial stromal sarcoma: report of three cases. *Path Int* **48**: 297–302

Gadducci A, Sartori E, Landoni F, Zola P, Maggino T, Urgesi A, Lissoni A, Losga G and Fanucci A (1996) Endometrial stromal sarcoma: analysis of treatment failures and survival. *Gynecol Oncol* **63**: 247–253

Gloor E, Schneyder P, Cikes M, Hofstetter J, Cordey R, Burnier F and Knobel P (1982) Endolymphatic stromal myosis. Surgical and hormonal treatment of extensive abdominal recurrence 20 years after hysterectomy. *Cancer* **50**: 1888–1893

Helin H, Isola J, Helle M and Koivula T (1990) Discordant results between radioligand and immunohistochemical assays for steroid receptors in breast carcinoma. *Br J Cancer* **62**: 109–112

Horowitz K, Rutherford T and Schwartz PE (1996) Hormone replacement therapy in women with sarcomas. *CME J Gynecol Oncol* **1**: 23–29

Katz L, Merino MJ, Sakamoto H and Schwarz PE (1987) Endometrial stromal sarcoma: a clinicopathologic study of 11 cases with determination of estrogen and progestin receptor levels in three tumors. *Gynecol Oncol* **26**: 87–97

Keen CE and Philip G (1989) Progesterone-induced regression in low grade endometrial stromal sarcoma. Case report and literature review. *Br J Obstet Gynaecol* **96**: 1435–1439

Koss LG, Spiro RH and Brunschwig A (1965) Endometrial stromal sarcoma. *Surg Gynecol Obstet* **121**: 531–537

Krieger PD and Gusberg SB (1973) Endolymphatic stromal myosis – a grade I endometrial sarcoma. *Gynecol Oncol* **1**: 299–313

Krumholz BA, Lobovsky FY and Halitsky V (1973) Endolymphatic stromal myosis with pulmonary metastases. Remission with progestin therapy: report of a case. *J Reprod Med* **10**: 85–89

Lantta M, Kahanpää K, Kärkkäinen J, Lehtovirta P, Wahlström T and Wildholm O (1984) Estradiol and progesterone receptors in two cases of endometrial stromal sarcoma. *Gynecol Oncol* **18**: 233–239

Luo S, Martel C, Gauthier S, Merand Y, Belanger A, Labrie C and Labrie F (1997) Long term effects of a novel anti-estrogen on the growth of ZR-75-1 and MCF-7 human breast cancer tumors in nude mice. *Int J Cancer* **73**: 735–739

Miller WR, Mullen P, Telford J and Dixon JM (1998) Clinical importance of intratumoral aromatase. *Breast Canc Res Treat* **49**: 27–32

Montag TW and Manart FD (1989) Endolymphatic stromal myosis: surgical and hormonal therapy for extensive venous recurrence. *Gynecol Oncol* **33**: 255–260

Navarro D, Cabrera JJ, Leon L, Chirino R, Fernandez L, Lopez A, Rivero JF, Fernandez P, Falcon O, Jimenz P, Pestano J, Diaz-Chico JC and Diaz-Chico BN (1992) Endometrial stromal sarcoma expression of estrogen receptors, progesteron receptors and estrogen-induced srp27 (24k) suggests hormone responsiveness. *J Steroid Biochem Mol Biol* **41**: 589–596

Pellillo D (1968) Proliferative stromatosis of the uterus with pulmonary metastases. Remission following treatment with a long acting synthetic progestin: a case report. *Obstet Gynecol* **31**: 33–39

Pickartz H, Beckmann R, Fleige B, Due W, Gerdes J and Stein H (1990) Steroid receptors and proliferative activity in non neoplastic and neoplastic endometria. *Virch Arch A Pathol Anat* **417**: 163–171

Piver MS, Rutledge FN, Copeland L, Webster K, Blumenson L and Suh O (1984) Uterine endolymphatic stromal myosis: a collaborative study. *Obstet Gynecol* **64**: 173–178

Press MF and Scully RE (1985) Endometrial ‘sarcomas’ complicating ovarian thecoma, polycystic ovarian disease and estrogen therapy. *Gynecol Oncol* **21**: 135–154

Rand RJ and Lowe JW (1990) Low-grade endometrial sarcoma treated with a progestagen. *Br J Hosp Med* **43**: 154–156

Remmele W and Schicketanz KH (1993) Immunohistochemical determination of estrogen and progesterone receptor content in human breast cancer: computer-assisted image analysis (QIC score) vs subjective grading (IRS). *Pathol Res Pract* **189**: 862–866

Richer JK, Lange CA, Wierman AM, Brooks KM, Tung L, Takimoto GS and Horwitz KB (1998) Progesterone receptor variants found in breast cells repress transcription by wild-type receptors. *Breast Cancer Res Treat* **48**: 231–241

Robertson JFR, Bates K, Pearson D, Blamery RWB and Nicholson RI (1992) Comparison of two oestrogen receptor assays in the prediction of the clinical course of patients with advanced breast cancer. *Br J Cancer* **65**: 727–730

© 2000 Cancer Research Campaign 

Sabini G, Chumas JC and Mann WJ (1992) Steroid hormone receptors in endometrial stromal sarcomas: a biochemical and immunohistochemical study. *Am J Clin Pathol* **97**: 381–386
Salmi A, Pakarinnen P, Peltola AM and Rutanen EM (1998) The effect of intrauterine levonorgestrel use on the expression of c-JUN, oestrogen receptors, progesterone receptors and Ki-67 in human endometrium. *Mol Hum Reprod* **4**: 1110–1115
Scribiner DR and Walker H (1998) Low-grade endometrial stromal sarcoma preoperative treatment with Depo-Lupron and Mecane. *Gynecol Oncol* **71**: 458–460
Scully RE, Bonfiglio TA, Kurman RJ, Silverberg SG and Wilkinson EJ (1994) *Histological Typing of Female Genital Tract Tumors*. Springer: Berlin
Styron SL, Burke TW and Linville WK (1989) Low-grade endometrial stromal sarcoma recurring over three decades. *Gynecol Oncol* **35**: 275–278
Sutton GP, Stehman FB, Michael H, Young PC and Ehrlich CE (1986) Estrogen and progesterone receptors in uterine sarcomas. *Obstet Gynecol* **68**: 709–714
Tosi P, Sforza V and Santoipietro R (1989) Estrogen receptor content, immunohistochemically determined by monoclonal antibodies in endometrial stromal sarcoma. *Obstet Gynecol* **73**: 75–78
Tsukamoto N, Kamura T, Matsukuma K, Imachi M, Uchino H, Saito T and Ono M (1985) Endolymphatic stromal myosis: a case with positive estrogen and progesterone receptors and good response to progestins. *Gynecol Oncol* **20**: 120–128
Vignali M and Genazzani L (1998) Molecular action of GnRH analogues on ectopic endometrial cells. *Gynecol Obstet Invest* **45**: 2–5

*© 2000 Cancer Research Campaign*
